# Improving Ambulatory Saliva-Sampling Compliance in Pregnant Women: A Randomized Controlled Study

**DOI:** 10.1371/journal.pone.0086204

**Published:** 2014-01-22

**Authors:** Julian Moeller, Roselind Lieb, Andrea H. Meyer, Katharina Quack Loetscher, Bettina Krastel, Gunther Meinlschmidt

**Affiliations:** 1 University of Basel, Department of Psychology, Division of Clinical Psychology and Epidemiology, Basel, Switzerland; 2 Diagnostic and Crisis Intervention Centre, Department of Psychiatry, University of Basel, Basel, Switzerland; 3 Department of Obstetrics, University Hospital Zurich, Zurich, Switzerland; 4 National Centre of Competence in Research (NCCR), Swiss Etiological Study of Adjustment and Mental Health (sesam), University of Basel, Basel, Switzerland; 5 Faculty of Medicine, Ruhr-University Bochum, Bochum, Germany; University of Warwick – Medical School, United Kingdom

## Abstract

**Objective:**

Noncompliance with scheduled ambulatory saliva sampling is common and has been associated with biased cortisol estimates in nonpregnant subjects. This study is the first to investigate in pregnant women strategies to improve ambulatory saliva-sampling compliance, and the association between sampling noncompliance and saliva cortisol estimates.

**Methods:**

We instructed 64 pregnant women to collect eight scheduled saliva samples on two consecutive days each. Objective compliance with scheduled sampling times was assessed with a Medication Event Monitoring System and self-reported compliance with a paper-and-pencil diary. In a randomized controlled study, we estimated whether a disclosure intervention (informing women about objective compliance monitoring) and a reminder intervention (use of acoustical reminders) improved compliance. A mixed model analysis was used to estimate associations between women's objective compliance and their diurnal cortisol profiles, and between deviation from scheduled sampling and the cortisol concentration measured in the related sample.

**Results:**

Self-reported compliance with a saliva-sampling protocol was 91%, and objective compliance was 70%. The disclosure intervention was associated with improved objective compliance (informed: 81%, noninformed: 60%), *F*(1,60)  = 17.64, *p*<0.001, but not the reminder intervention (reminders: 68%, without reminders: 72%), *F*(1,60)  = 0.78, *p* = 0.379. Furthermore, a woman's increased objective compliance was associated with a higher diurnal cortisol profile, *F*(2,64) = 8.22, *p*<0.001. Altered cortisol levels were observed in less objective compliant samples, *F*(1,705) = 7.38, *p* = 0.007, with delayed sampling associated with lower cortisol levels.

**Conclusions:**

The results suggest that in pregnant women, objective noncompliance with scheduled ambulatory saliva sampling is common and is associated with biased cortisol estimates. To improve sampling compliance, results suggest informing women about objective compliance monitoring but discourage use of acoustical reminders.

## Introduction

Maternal stress during pregnancy can adversely affect birth outcomes and offspring development (e.g. [1], [2]). Cortisol as a stress marker, released by the maternal hypothalamic–pituitary–adrenal (HPA) axis, may partly explain these effects (for reviews see [3]–[6]). Therefore, psychoneuroendocrine research examining cortisol in pregnant women has been given high priority and may contribute to a better understanding of the biochemical mechanism underlying the adverse effects described above [4]. Research examining cortisol in pregnant women may also improve risk assessment for adverse birth outcomes [7], [8].

In ambulatory settings, cortisol concentrations are commonly assessed noninvasively in saliva. Subjects are instructed to collect a certain number of saliva samples at scheduled sampling times on one or more consecutive study days [9]–[11]. Compared to laboratory research, ambulatory research results in higher ecological validity [12]. However, ambulatory saliva sampling may be biased by noncompliance: Subjects may not follow scheduled sampling times and may fail to self-report this noncompliance in paper-and-pencil diaries – even if study collaborators stress the importance of both requirements. Indeed, such patterns have been observed in studies using hidden electronic compliance-monitoring systems, comparing subjects' objective compliance to their self-reported compliance [13]–[15]. Noncompliance with scheduled saliva-sampling times has been associated with biased cortisol estimates due to the cortisol circadian rhythm [13], [14], [16]. Biased estimates of cortisol concentrations may cause invalid interpretations of the data. In contrast, Jacobs et al. [15] reported no biased cortisol estimates when noncompliant saliva samples were included in analyses. Saliva-sampling noncompliance has also been associated with additional study costs and, in the case of missing samples, with reduced statistical power [17].

To deal with the compliance problem, experimental studies tested whether informing subjects about objective compliance monitoring improves compliance with scheduled saliva sampling. In these studies, subjects who were informed about monitoring displayed higher compliance with the sampling protocols compared to noninformed subjects [13], [14]. Acoustical reminders such as preprogrammed wristwatches have been used to improve saliva sampling compliance [18], [19], although, to our knowledge, their effect on saliva-sampling compliance has not yet been experimentally tested, and experimental evidence that they improve compliance comes from other research fields only: For example, in a review, reminders improved medication compliance in antiretroviral therapy in four of eight studies [20]. Moreover, electronic reminders improved participants' compliance with paper pain diaries, but still, according to Broderick and colleagues [21], the compliance rates were unsatisfactory.

In sum, ambulatory saliva sampling has gained great importance in psychoneuroendocrine research, being used to examine cortisol concentrations in pregnant women. However, findings that noncompliance with saliva sampling is common and can bias cortisol estimates, and that informing subjects about objective compliance monitoring improves saliva-sampling compliance, are based on ambulatory research in healthy volunteers, patients, and an older population [13], [14], [16]. It is unclear whether these findings can be generalized to pregnant women, especially as pregnant women display different behavioral patterns (e.g. reduced physical activity, more sitting, lying, sleeping, and slower walking pace) [22]–[24], altered cortisol levels, and altered cortisol responses to stress [5], [25], [26] compared to nonpregnant controls or to nonpregnant state. To our knowledge, it has not yet been investigated in a sample of pregnant women whether informing them about objective compliance monitoring improves compliance with scheduled saliva sampling, or whether noncompliance biases cortisol estimates. Moreover, to our knowledge, whether acoustical reminders improve saliva-sampling compliance has not yet been experimentally tested at all.

The goals of the present study were a) to estimate compliance rates with a standard ambulatory saliva-sampling protocol; b) to estimate whether the strategies of informing subjects about objective compliance monitoring and using acoustical reminders improve compliance with scheduled saliva sampling; and c) to estimate the association between saliva-sampling noncompliance and saliva cortisol concentrations in pregnant women.

## Methods

### Ethics Statement

The study was conducted in accordance with the Declaration of Helsinki and approved by the ethics committees of Basel (Ethikkommission beider Basel, Basel, Switzerland) and Zurich (Kantonale Ethikkommission Zuerich, Zuerich, Switzerland). All participants gave written informed consent.

### Subjects

We recruited pregnant women during antenatal visits at the outpatient service of the Department of Obstetrics, University Hospital Zurich, Switzerland. Eligible women were in their 12^th^ to 32^nd^ week of gestation, had sufficient German language skills, and underwent regular antenatal visits at the outpatient service. Exclusion criteria were diseases potentially affecting the neuroendocrine system, high-risk pregnancy, human immunodeficiency virus (HIV) infection, and the use of hormone-containing medication. These criteria were chosen to minimize distortions in the women's cortisol concentrations.

### Experimental interventions and design

We told all the women to collect eight saliva samples on two consecutive days. Scheduled sampling times were: 0, 30, 45, and 60 min after awakening, and at 1100, 1500, 2000, and 2200 h. Objective compliance with the time of sampling was monitored, in all women, with a hidden Medication Event Monitoring System (MEMS 6 TrackCap Monitor, Aardex Ltd., Switzerland). Two interventions were tested by creating four experimental groups: Group 1 received information about the objective compliance monitoring at the beginning of the study and received timers (Kuechentimer, Zyliss, Switzerland) and alarm clocks (basic alarm clock, Intertronic, Switzerland) to provide acoustical reminders at the scheduled sampling times. The second group received only the information about the objective compliance monitoring. The third group received the acoustical reminders alone, and the fourth group received neither the information about the objective compliance monitoring nor acoustical reminders. Women in the groups receiving the acoustical reminders were advised to use the timer to time the samples at +30, +45, and +60 min after awakening and the clock to time the 1100 h, 1500 h, 2000 h, and 2200 h samples, even if they possessed their own watch.

In sum, a randomized 2 (disclosure intervention: informed vs. noninformed) ×2 (reminder intervention: acoustical reminders vs. no acoustical reminders) ×2 (days) ×8 (sampling times) design was applied. A blocked randomization sequence was created with a computerized random number generator and applied with a 1:1:1:1 assignment to the four experimental groups. Study collaborators using sealed envelopes conducted the assignment. All women were blinded to the true nature of the present study; laboratory staff analyzing the saliva samples were blinded to the women's experimental group assignment.

### Procedure

During an antenatal visit, obstetricians told women who met the inclusion criteria about the study. A study collaborator gave standardized information to interested women. Women who agreed to participate were assigned to the experimental groups as described above and received packages with the respective study materials. A study collaborator, stressing the importance of high compliance with the sampling protocol, explained the use of the material in detail. The women were instructed to collect saliva samples on two consecutive days right before their next scheduled antenatal visit. Three days before sampling, a study collaborator contacted them by telephone as a reminder and to answer any questions. The women provided demographic information, including age, height, employment status, number of hours worked per week, prepregnancy body weight, current body weight, gestational age of their fetus, gravidity, and parity, via questionnaire. They were instructed not to brush their teeth, eat, consume caffeine, or smoke during the first hour after awakening and 1 h before each scheduled saliva sampling. They were also advised to avoid physical exercise but to otherwise follow their daily routine. These restrictions were meant to minimize distortions in the women's cortisol concentrations. The women handed over the study material to a study collaborator the day after sampling, at the antenatal visit. On this occasion, we asked the women, by questionnaire, whether there was anything that had attracted particular attention or was particularly noticeable during the study. In doing so, we sought to test whether noninformed women obtained knowledge about objective compliance monitoring. Finally, the women were debriefed about the true nature of the study.

### Saliva sampling

Straws and 2.0-mL safe-lock tubes (Eppendorf, Hamburg, Germany), labeled with scheduled sampling time, were clearly arranged until usage in a transparent MediDispenser (Wiegand, Buelach, Switzerland). Women were instructed to place the tubes immediately after saliva sampling into small nontransparent medicine containers (Wiegand, Buelach, Switzerland) fitted with MEMS 6 caps. As a cover story, all the women were told that this procedure was important to maintain sample quality by minimizing light exposure. For the same reason, they were advised to open the medicine container only to insert the saliva samples. This container was to be stored overnight and, when possible, in a refrigerator.

### Biochemical analyses

We froze returned saliva samples at −20°C until biochemical analysis. Thawed samples were centrifuged at 3000 g for 10 min. Salivary free cortisol was analyzed using a commercial enzyme immunoassay for human saliva (cortisol ELISA, IBL, Hamburg, Germany). Analytical assay sensitivity was 2.0 pg/mL. The intra- and interassay coefficients of variation were ≤7.3% and ≤9.3%, respectively.

### Compliance with saliva sampling

We assessed self-reported and objective compliance with scheduled saliva-sampling times. Self-reported compliance was assessed with a paper-and-pencil diary, in which the women were asked to record the exact time and date of each saliva sampling. Objective compliance was assessed with the MEMS 6 caps that recorded the moment of each opening and closing of the medical container. The opening times of the MEMS 6 caps were processed with PoverView (Aardex Ltd., Switzerland). Compliance criteria were adapted from Kudielka et al. [14] and applied for both self-report and objective compliance. Accordingly, we classified the +0-min sample as compliant if collected within ±10 min of the self-reported wake-up time, the +30-, +45-, and +60-min samples as compliant if collected within ±7 min, and the 1100 h, 1500 h, 2000 h, and 2200 h samples as compliant if collected within ±1 h of the scheduled sampling time. In the case of multiple MEMS 6 cap openings around the scheduled sampling times, we selected the most compliant. If a women delivered more saliva samples than recorded MEMS 6 cap opening times, we selected the most compliant opening times for the delivered samples and classified the remaining samples as noncompliant.

### Statistical analyses

In the first set of analyses, we estimated the association of the two interventions with objective compliance using general linear models (GLMs). Disclosure intervention (informed vs. noninformed) and reminder intervention (acoustical reminders vs. no acoustical reminders) were the two fixed independent factors, and objective compliance (percentage of compliant samples) was the dependent variable. The +0-min samples were excluded, as we could not objectively determine whether the women reported their wake-up times accurately. We repeated the analysis described above with objective morning compliance (+30-, +45-, and +60-min samples only; percentage of compliant samples) as the dependent variable. High compliance with the morning samples is considered especially important as the cortisol awakening response (CAR) is often used for research purposes as indicator of HPA reactivity [11]. The CAR represents the rapid steep increase of cortisol concentrations within the first 30 min of awakening [11], [27].

In the second set of analyses, we estimated the association of objective compliance with saliva cortisol concentrations using a random coefficient model, a type of linear mixed model [28]. This type of model has been shown to provide more efficient and less biased results in data where missing values occur, compared with complete case analyses or analyses in which missing values are imputed using the last observation carried forward method [29]. Further, linear mixed models do not require omitting subjects with missing data from the analyses, thereby minimizing data loss and risk of bias while increasing power. Our model included a random intercept as well as a random slope parameter when this improved model fit (based on Akaike's Information Criterion, AIC) [28].

The random coefficient model allowed us to differentiate between objective state and trait compliance. Trait compliance is a time-invariant predictor and measuring it allowed us to estimate whether the women's objective compliance with the sampling schedule was associated with their diurnal profiles of cortisol concentrations. We estimated the effect of the women's objective compliance by dividing the women into a low (0–5 compliant samples; 0–31% of all scheduled samples), a moderate (6–12 compliant samples; >31–80%), and a high (13–16 compliant samples; >80–100%) compliance group. We used the categorical compliance predictor instead of the continuous compliance predictor “number of compliant samples” because preliminary analyses revealed better model fits for the former. For the high compliance group, we chose the 80% cut-off because prior research used this cut-off to classify compliance in a cortisol-sampling protocol [30] and because medical research usually classifies patients with compliance of more than 80% as compliant [30], [31]. To enlarge the small sample size in the low compliance group, we chose a 31% cut-off (0–5 compliant samples) instead of the 20% cut-off applied by Hall et al. [30]. For the predictor time we assumed linear trajectories for each of two intervals covering the time points +0- to +30-min and +30-min to the last time point (2200 h), respectively.

State compliance relates to individual saliva samples and allowed us to estimate whether a deviation from a scheduled sampling time was associated with the cortisol concentration measured in the related sample. We estimated this association by entering the time-varying predictor “deviation from scheduled sampling time in minutes” into our model. Again, we excluded the +0-min samples for the same reasons as stated above. The predictor time was again assumed to be linear, but we only considered the interval +30-min to 2200 h. We repeated our mixed model analyses, adjusting for several a priori defined potential time-invariant confounders, including the continuous covariates age, gestational age of fetus, parity [32], and current body weight [33].

The percentage of compliant samples was arcsine transformed, cortisol data were square root transformed, and deviations from scheduled sampling times in minutes were log transformed to approximate normal distributions. An alpha level of 0.05 determined statistical significance. Data analysis was carried out using IBM SPSS Statistics 20 for Mac OS X.

## Results

We included 75 eligible women in the present study. Six women declined further participation before saliva sampling. Two women were excluded because of a MEMS 6 cap defect and two because they collected saliva samples without using the MEMS 6 caps. Another woman was excluded because she took part only on the first day of the study because she delivered prematurely on the second day. Thus, the final sample consisted of 64 women. Demographic information is presented in Table 1. None of the noninformed women reported any knowledge of the objective compliance monitoring on the questionnaire before the debriefing.

**Table 1:** Demographic variables^a^ across experimental groups.

Variable	Total	Compliance monitoring			
	(*n* = 64)	Informed		Noninformed	
		Acoustical reminders		Acoustical reminders	
		With	Without	With	Without
		(*n* = 16)	(*n* = 17)	(*n* = 15)	(*n* = 16)
Age (years)	33 (28;36)	33 (27;39)	33 (28;35)	34 (32;40)	31 (26;35)
Height (cm)	167 (163;172)	163 (162;172)	164 (161;170)	170 (166;173)	168 (165;172)
Employed^b^					
Yes	45 (70.3)	10 (62.5)	14 (82.4)	9 (60)	12 (75)
No	16 (25)	5 (31.3)	2 (11.8)	5 (33.3)	4 (0)
Unknown	3 (4.7)	1 (6.3)	1 (5.9)	1 (6.7)	0 (0)
Hours worked per week	28 (17;42)	35 (11;42)	25 (15;41)	40 (20;51)	28 (24;42)
Prepregnancy body weight (kg)	60 (56;68)	59 (56;71)	59 (54;64)	61 (58;69)	59 (55;67)
Current body weight (kg)	70 (62;75)	67 (63;76)	68 (58;74)	71 (64;85)	72 (60;75)
Gestational age of fetus	26 (17;31)	26 (16;30)	22 (17;31)	29 (21;34)	24 (17;29)
Gravidity^b^					
0	28 (43.8)	7 (43.8)	6 (35.3)	6 (40)	9 (56.3)
1–2	21 (32.8)	4 (25)	6 (35.3)	5 (33.4)	6 (37.5)
≥3	9 (14.2)	2 (12.6)	3 (17.7)	3 (20)	1 (6.3)
Unknown	6 (9.4)	3 (18.8)	2 (11.8)	1 (6.7)	0 (0)
Parity^b^					
0	34 (53.1)	9 (56.3)	8 (47.1)	7 (46.7)	10 (62.5)
1–2	21 (32.8)	3 (18.8)	6 (35.3)	6 (40)	6 (37.5)
≥3	3 (4.7)	1 (6.3)	1 (5.9)	1 (6.7)	0 (0)
Unknown	6 (9.4)	3 (18.8)	2 (11.8)	1 (6.7)	0 (0)

a If not otherwise specified, median (25 percentile; 75 percentile) is reported.

b Number of pregnant women (percent) is reported.

### Compliance with the saliva-sampling protocol and interventions

Self-reported compliance and objective compliance refer to all samples (+30-, +45-, +60-min, 1100 h, 1500 h, 2000 h, and 2200 h samples), and objective morning compliance refers to the +30-, +45-, and +60-min samples only. Across all the women, self-reported compliance with the saliva-sampling protocol was 91% and objective compliance was 70%. Self-reported compliance was high in all experimental groups (range 88–94%). Objective compliance was highest in the informed group without acoustical reminders (86%) and lowest in the noninformed group without acoustical reminders (58%). The women's objective morning compliance was lower (59%) compared to their objective compliance reported above (70%). Moreover, self-reported compliance in women with low objective compliance (0–31% compliant samples) was 80%. Descriptive compliance data are presented in Table 2.

**Table 2:** Self-reported and objective compliance with scheduled saliva sampling across experimental groups.

Descriptive data	Self-reported compliance					Objective compliance				
	Informed		Non-informed		Total	Informed		Non-informed		Total
	Acoustical reminders		Acoustical reminders			Acoustical reminders		Acoustical reminders		
	With	Without	With	Without		With	Without	With	Without	
	(*n* = 15)	(*n* = 16)	(*n* = 14)	(*n* = 15)	(*n* = 60)^a^	(*n* = 16)	(*n* = 17)	(*n* = 15)	(*n* = 16)	(*n* = 64)
**Morning samples** b										
Number of scheduled samples	90	96	84	90	360	96	102	90	96	384
Number of compliant samples	73	88	76	74	311	60	76	43	46	225
Compliant samples in % (SD)	81% (25.9)	92% (25.1)	90% (18.2)	82% (30.5)	86% (25.2)	62% (24.0)	75% (25.1)	48% (28.8)	48% (30.3)	59% (28.8)
**All samples** c										
Number of scheduled samples	210	224	196	210	840	224	238	210	224	896
Number of compliant samples	185	210	177	192	764	168	204	129	130	631
Compliant samples in % (SD)	88% (13.4)	94% (12.2)	90% (21.3)	91% (13.5)	91% (15.1)	75% (20.2)	86% (16.4)	61% (25.6)	58% (25.5)	70% (24.3)
**Summed absolute deviations from scheduled sampling times in minutes** c										
Median	34	51	53	25	47	145	92	248	185	147
25th percentile; 75th percentile	12; 120	28; 92	40; 107	0; 80	15; 99	60, 281	56, 190	147; 553	87; 369	74; 274

a Number in sample for self-reported compliance is four less than number in sample for objective compliance because of missing data in self-report questionnaires.

b Including the +30-min, +45-min, and +60-min samples on two consecutive days.

c Including the +30-min, +45-min, +60-min samples and the 1100 h, 1500 h, 2000 h, and 2200 h samples on two consecutive days.

SD, standard deviation.

Objective compliance in informed and noninformed women was 81% and 60%, and in women with and without acoustical reminders 68% and 72%, respectively. The GLM showed significant main effects of disclosure intervention (informed vs. noninformed) on both objective compliance, *F*(1,60)  = 17.64, *p*<0.001, and objective morning compliance, *F*(1,60)  = 9.27, *p* = 0.003. However, there was no significant main effect of reminder intervention (acoustical reminders vs. no acoustical reminders) on either compliance type [objective compliance, *F*(1,60)  = 0.78, *p* = 0.379; objective morning compliance, *F*(1,60)  = 0.80, *p* = 0.374]. Interaction effects between disclosure intervention and reminder intervention were also nonsignificant for both compliance types [objective compliance, *F*(1,60)  = 2.46, *p* = 0.122; objective morning compliance, *F*(1,60)  = 0.77, *p* = 0.385].

### Objective compliance and cortisol concentrations

Objective compliance information was used to estimate the associations between compliance with scheduled sampling and cortisol concentrations.

#### Cortisol concentrations in high-, moderate-, and low-compliance women

**Figure 1:**
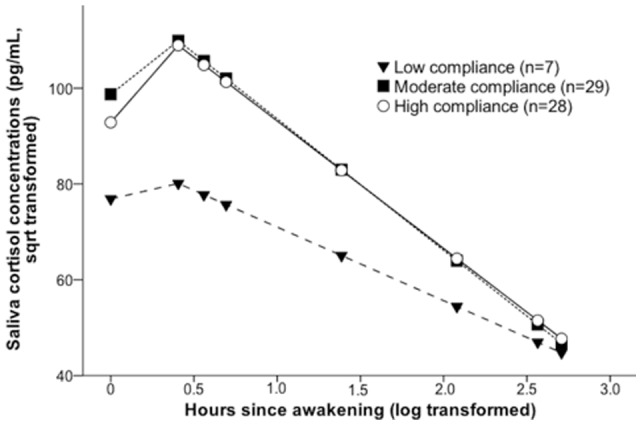
Cortisol concentrations in women with low, moderate, and high compliance. Saliva cortisol concentrations were averaged across two sampling days representing estimated values from a linear mixed model.

Twenty-eight women (44%) showed high objective compliance with the saliva-sampling protocol, 29 (45%) moderate objective compliance, and seven (11%) low objective compliance. Using random coefficient models, we compared the cortisol concentrations of women with high, moderate, and low objective compliance. We found a main effect for objective compliance on cortisol concentrations, *F*(2,64)  = 8.22, *p*<0.001, which was due almost entirely to the difference in cortisol concentrations between objective low-compliance women on the one hand and the combined objective moderate- and high-compliance women on the other, *F*(1,74)  = 16.14, *p*<0.001, for contrast. However, there was no difference in cortisol concentrations between objective moderate- and high-compliance women, *F*(1,63)  = 0.41, *p* = 0.525. We also found an interaction effect between objective compliance and time of saliva measurement on cortisol concentrations, *F*(2,64)  = 5.26, p = 0.008. As shown in Figure 1, cortisol levels of women in the low-compliance group were lower than cortisol levels of women in the moderate- and high-compliance groups, but this effect slowly disappeared throughout the day, such that at the 2200 h scheduled sampling time, women at the three levels of compliance displayed comparable cortisol levels. Accordingly, if we considered morning cortisol concentrations only (+0-, +30-, +45-, and +60-min samples), we still found a main effect of objective compliance on morning cortisol concentrations, *F*(2,64)  = 10.24, *p*<0.001. Intraindividual variation in these morning concentrations is indicative of the CAR. The associations stated above did not change significantly after adjustment for age, current body weight, gestational age of fetus, and parity (data available on request).

#### Deviation from scheduled sampling and the cortisol concentration in the related sample

A total of 753 saliva samples were included in this analysis. An objective deviation from a scheduled sampling time was associated with the cortisol level measured in the related sample, *F*(1,705)  = 7.38, *p* = 0.007; that is, the longer the time delay from a scheduled sampling, the lower the cortisol level. The cortisol levels (on the square-root scale) decreased per minute deviation (on the natural logarithm scale) by a value of 0.82 (SE 0.30). The nonsignificant interaction effect between deviation from scheduled sampling time and time of saliva measurement on cortisol concentrations, *F*(1,713)  = 2.50, *p* = 0.115, indicated a stable association of deviation from scheduled sampling time with cortisol concentrations throughout the day. The associations stated above did not change significantly after adjustment for age, current body weight, gestational age of fetus, and parity (data available on request).

## Discussion

Objective noncompliance with scheduled saliva sampling was associated with biased cortisol estimates in pregnant women. Informing women about the compliance monitoring improved objective compliance with scheduled saliva sampling. In contrast, the use of acoustical reminders had no effect on objective compliance.

### Compliance with the saliva-sampling protocol

The women's self-reported compliance was higher than their objective compliance with the saliva-sampling protocol, especially when they were not informed about the compliance monitoring. Even the women with low objective compliance (0–31% compliant samples) self-reported, on average, high compliance. These findings are in line with prior studies in nonpregnant subjects showing a possible bias in self-reported saliva-sampling compliance [13]–[15].

In the present study, women informed about compliance monitoring displayed objective compliance rates of 75% (with acoustical reminders) and 86% (without acoustical reminders). By comparison, prior studies found objective compliance rates in informed subjects of 89–97% [13], [14]. Objective compliance rates in our noninformed women were 61% (with acoustical reminders) and 58% (without acoustical reminders). In contrast, noninformed subjects in prior studies had objective compliance rates of 62–84% [13], [14]. Several reasons may explain our somewhat lower objective compliance rates compared to the prior studies: First, there may be specific behavioral patterns in pregnant women [22]–[24]. Second, there is the issue of saliva-sampling burden. Kudielka et al. [16] hypothesized that a higher sampling burden related to the number of scheduled saliva samples per day may lead to lower saliva-sampling compliance. In line with this, the daily saliva-sampling burden in the present study (eight samples per day) was higher than that in the studies of Kudielka et al. (six samples per day; [14]) and Broderick et al. (five samples per day; [13]). Third, other differences in study design may account for lower compliance rates in the present study (e.g. we applied more conservative compliance criteria regarding the morning samples compared to Broderick et al.) [13]. Moreover, we cannot exclude further factors in pregnant women associated with lower objective saliva-sampling compliance.

### Associations of the interventions with objective compliance

In this randomized controlled trial, the disclosure intervention (informing about compliance monitoring) was associated with higher objective compliance with the saliva-sampling protocol. This finding is in line with prior studies in nonpregnant subjects [13], [14] and suggests that informing about compliance monitoring improves saliva-sampling compliance in pregnant women. Thus, the present study extends earlier results to a sample of pregnant women. In the present study, informing the women was associated with higher objective compliance with respect to both all scheduled samples and the scheduled morning samples. High compliance with the morning samples is particularly relevant because the CAR has been used extensively as an indicator of HPA activity, and because the CAR is increasingly relevant in endocrine research in pregnant women (e.g., [7], [26], [34], [35]).

In contrast, we did not find any positive effect of the reminder intervention on objective compliance. Using acoustical reminders was not associated with improved objective saliva-sampling compliance. One possible explanation for this observation is that carrying timers and alarm clocks was rather inconvenient. Indeed, several women reported this during the debriefing at the end of the study. To our knowledge, the present study is the first to investigate the association of acoustical reminders with saliva-sampling compliance. However, in the research field of antiretroviral therapy in HIV treatment, a review described that the use of electronic reminders improved medication compliance in four of eight studies [20]. This review, however, included studies relying on self-report measures. In a recent randomized controlled trial, the use of pocket digital alarms had no effect on objective medication compliance, as measured by the percentage of dispensed drug doses [36]. Thus, in line with the latter, our data discourages the use of acoustical reminders to improve saliva-sampling compliance in pregnant women: While having no positive effect on compliance, the use of acoustical reminders increases the study burden on women and generates additional study costs.

### Associations of objective compliance with cortisol concentrations

The women's objective compliance with the saliva-sampling protocol was associated with their cortisol concentrations. Women with low compliance displayed lower cortisol levels compared to those with moderate or high compliance. In detail, women with low compliance showed lower CARs and downward-shifted day slopes of cortisol compared to women with moderate and high compliance. One explanation could be that women with low compliance deliver samples more often with a delay, which – due to the diurnal decline of cortisol concentrations – is likely to be associated with lower cortisol levels, resulting in lower levels on average. Alternatively, being low compliant may be related to certain trait characteristics, which in turn may be associated with lower cortisol levels. Cortisol levels did not differ between women with moderate and high compliance. Hence, low compliance may bias cortisol estimates, but moderate compliance may have less impact. The finding that noncompliance may bias cortisol results is in line with prior studies in nonpregnant subjects [13], [14], [16].

Without objective compliance monitoring, we would not have been able to identify the biased cortisol estimates of women with low compliance, as they incorrectly self-reported high compliance. Thus, without objective compliance information, cortisol slopes or CARs, biased by low compliance, could lead to invalid interpretations. For example, prior research has associated lower morning cortisol levels with cumulative stress in pregnant women [37]. Without objective compliance information, it might be difficult to conclude whether lower morning cortisol levels are directly associated with cumulative stress or with a bias introduced by saliva-sampling noncompliance related to stress (compare [14]). In the present study, the association between women's objective compliance and their cortisol levels decreased through the day. This finding could be important as prior research used late afternoon or evening cortisol data instead of the CAR to examine specific research questions in pregnant women [38], [39]. Based on our findings, when objective compliance information is not available, using evening samples may reduce the potential bias in cortisol estimates introduced by noncompliance.

In the present study, women's compliance was associated with their diurnal profiles of cortisol concentrations. Moreover, we observed altered cortisol levels in less-compliant samples. The larger a time delay from a scheduled saliva sampling, the stronger was the bias by reduced cortisol levels. This finding confirms the importance of saliva-sampling compliance in pregnant women.

### Limitations and strengths

The present study has several limitations. First, the sample size of our low-compliance group was small. Hence, our findings should be replicated in a larger sample. Second, the following hampered calculating a priori power analyses: To our knowledge, the present study is the first to estimate whether acoustical reminders improve saliva-sampling compliance; hence, estimation of effect sizes was difficult. Moreover, power analysis for mixed models requires information regarding estimates of intraclass correlations, which were not available in our case. Third, we could not control whether the women actually used the acoustical reminders. Thus, the nonsignificant association between reminder intervention and saliva-sampling compliance could be due to women not having applied the intervention (compare [36]). However, we were less interested in whether the use of acoustical reminders improved compliance, but mainly interested in whether the distribution of acoustical reminders and the recommendation of their use improved compliance. Fourth, without actigraph monitoring, we could not objectively define whether women reported their wake-up times accurately. However, evidence suggests that self-reported wake-up times are reasonably accurate, compared with objectively measured wake-up times [40], [41]. Fifth, women may have collected saliva samples at scheduled times without storing them in the MEMS container. Putting several compliant saliva samples into the MEMS container at the same time would have led to missing MEMS 6 cap opening times and, thus, to objective compliant samples being classified as noncompliant. In the present study, this would have led to an underestimation of objective compliance rates. Last, findings regarding acoustical reminders may not be generalizable to other reminder systems. Further studies might examine whether other reminder systems (e.g. handheld computers, mobile apps) improve saliva-sampling compliance.

Despite the study's limitations, the present study has important strengths. First, we applied a standard two-day ambulatory saliva-sampling protocol, and second, we used a randomized controlled trial to test strategies to improve saliva-sampling compliance. Third, we used mixed model analyses to estimate the associations between compliance and cortisol concentrations. Mixed model analysis is considered the method of choice for analyzing ambulatory saliva cortisol data [11].

## Conclusions

Our study findings indicate that informing about the use of objective compliance monitoring substantially improved saliva-sampling compliance in pregnant women. In contrast, using acoustical reminders had no positive effect. They should inform future studies examining cortisol in pregnant women within ambulatory saliva-sampling designs and are highly important for several reasons. First, noncompliance with a standard ambulatory saliva-sampling protocol was common in pregnant women and occurred more frequently than in prior studies with nonpregnant subjects. Second, noncompliant women could not be identified by self-report data. Third, objective noncompliance biased estimates of women's cortisol concentrations and, hence, may have led to invalid interpretations. Thus, the present study encourages using objective compliance monitoring to identify noncompliance with a saliva-sampling protocol in pregnant women. Moreover, it suggests informing women about objective compliance monitoring to improve compliance.
